# Steroid receptor expression in the fish inner ear varies with sex, social status, and reproductive state

**DOI:** 10.1186/1471-2202-11-58

**Published:** 2010-04-30

**Authors:** Karen P Maruska, Russell D Fernald

**Affiliations:** 1 Biology Department, Stanford University, Stanford, CA., USA

## Abstract

**Background:**

Gonadal and stress-related steroid hormones are known to influence auditory function across vertebrates but the cellular and molecular mechanisms responsible for steroid-mediated auditory plasticity at the level of the inner ear remain unknown. The presence of steroid receptors in the ear suggests a direct pathway for hormones to act on the peripheral auditory system, but little is known about which receptors are expressed in the ear or whether their expression levels change with internal physiological state or external social cues. We used qRT-PCR to measure mRNA expression levels of multiple steroid receptor subtypes (estrogen receptors: ERα, ERβa, ERβb; androgen receptors: ARα, ARβ; corticosteroid receptors: GR2, GR1a/b, MR) and aromatase in the main hearing organ of the inner ear (saccule) in the highly social African cichlid fish *Astatotilapia burtoni*, and tested whether these receptor levels were correlated with circulating steroid concentrations.

**Results:**

We show that multiple steroid receptor subtypes are expressed within the main hearing organ of a single vertebrate species, and that expression levels differ between the sexes. We also show that steroid receptor subtype-specific changes in mRNA expression are associated with reproductive phase in females and social status in males. Sex-steroid receptor mRNA levels were negatively correlated with circulating estradiol and androgens in both males and females, suggesting possible ligand down-regulation of receptors in the inner ear. In contrast, saccular changes in corticosteroid receptor mRNA levels were not related to serum cortisol levels. Circulating steroid levels and receptor subtype mRNA levels were not as tightly correlated in males as compared to females, suggesting different regulatory mechanisms between sexes.

**Conclusions:**

This is the most comprehensive study of sex-, social-, and reproductive-related steroid receptor mRNA expression in the peripheral auditory system of any single vertebrate. Our data suggest that changes in steroid receptor mRNA expression in the inner ear could be a regulatory mechanism for physiological state-dependent auditory plasticity across vertebrates.

## Background

The peripheral and central auditory system of vertebrates is sensitive to sex- and stress-related steroid hormones, which can have profound effects on how an animal perceives acoustic information and ultimately behaves during social interactions. While steroids such as estradiol are known to influence hearing in many vertebrate taxa [1-4], the cellular and molecular mechanisms responsible for steroid-mediated auditory plasticity at the level of the inner ear remain unknown.

In mammals, females often have "better" hearing (e.g., improved high frequency hearing; shorter auditory brainstem response wave latencies) and later-onset of age-related hearing loss compared to males, as well as changes in hearing ability associated with the ovarian cycle and pregnancy [1]. Further, postmenopausal women who are on estrogen-based hormone replacement therapy (HRT) have better hearing than those who are not [1], while progestin-based HRT can diminish hearing ability [5,6]. These sex and female ovarian cycle variations in auditory ability are attributed to the protective effects of estrogen and may be partially related to estrogen receptor (ER) expression in the cochlea. This idea is supported by studies that demonstrate ERβ knockout mice that have profound hearing impairment and hair cell loss [7], and changes in ERα abundance in the ear that are correlated with fluctuating estrogen levels during pregnancy in rats [8]. A role for estrogen in hearing also extends to non-mammalian vertebrates. In female midshipman fish (*Porichthys notatus*), for example, estradiol shifts the tuning of primary afferent neurons that innervate the main hearing organ (saccule) possibly to better encode the higher frequency components of the male's advertisement call during the breeding season [3,9]. Moreover, ERα and aromatase, an enzyme necessary for estrogen biosynthesis, are found in the inner ear, which suggests direct action of estradiol at the periphery [3,10,11]. In contrast to these well-known effects of estradiol on hearing function [12], relatively little is known about how androgens might influence hearing (but see [3,13,14]) or whether androgen receptors (AR) are also expressed within the inner ear of vertebrates.

Physical or emotional stress and the subsequent release of glucocorticoid hormones (i.e., cortisol or corticosterone) can also regulate auditory sensitivity and inner ear homeostasis [15-17]. Moreover, glucocorticoids are used to treat several hearing disorders in humans such as hearing loss, tinnitus, Meniere's disease and autoimmune disease because of their quick (time course of seconds to hours) therapeutic response in the inner ear [1,18]. Glucocorticoid (GR) and mineralocorticoid receptors (MR) that respond to both cortisol and aldosterone are found in the inner ear of several mammalian species (i.e., mice, rats, guinea pigs, humans) [19-21], but have not yet been described in other vertebrate taxa. The conserved function of cortisol in the stress response system suggests a possible similar hypothalamic-pituitary-adrenal (or interrenal)-hearing link in non-mammalian vertebrates.

For steroids to influence hearing at the periphery, steroid receptors must be present in the inner ear, but few studies have measured steroid receptor expression in the vertebrate ear. Sex and/or stress-related steroid receptors have been localized to the inner ear in one fish [10,11,22], one bird [23], and several mammals [1], but little is known about how steroid receptor expression in the ear varies between the sexes within a species or across different reproductive states within a sex. Many vertebrates, especially teleost fishes, express multiple subtypes of a particular steroid receptor (i.e., ERα and ERβa/b, ARα and ARβ, GR2 and GR1a/b), and the distribution and abundance of these different forms within a tissue are known to be important determinants of how estrogen, androgen, and corticosteroid hormones function [24]. Our current understanding of the prevalence of multiple ER, AR, and CR isoforms within the ear of a single vertebrate species, however, is incomplete. Indeed, only recently have investigators begun to study changes in receptor expression as a potential mechanism for auditory plasticity [8,25]. To fully understand how steroid hormones might influence vertebrate inner ear function, we measured the expression patterns of all known ERs, ARs, and CRs in a single species, along with circulating gonadal and stress hormone levels.

**Figure 1:**
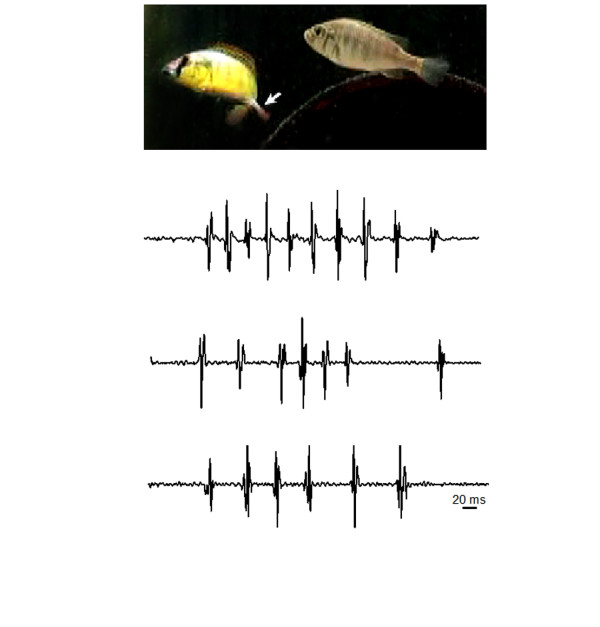
**Sound production during male courtship behavior in the African cichlid fish *Astatotilapia burtoni***. Dominant territorial males (fish at left) produce sounds that consist of ~4-12 short (~15-20 ms) pulses during body quivering while they present their anal fin egg-spots (arrow) towards a female (fish at right). Courtship sounds are always accompanied by male quivering behavior, but not all quivers are associated with sound production, which supports the hypothesis of intentional sound production for communication. Representative waveforms of individual courtship sounds from three different dominant territorial males are shown. Recordings were made with a calibrated hydrophone (High Tech, Inc.; sensitivity -163.7 dB re: 1 V/μPa; frequency response 2 Hz-30 kHz) suspended in the tank above the spawning territory.

Cichlid fishes use multiple sensory cues (i.e., visual, olfactory, auditory, mechanosensory) to coordinate their complex social behaviors, and show great diversity in reproductive and parental care strategies, which makes them excellent models for analyzing how hormones influence sensory function. We examined steroid receptor expression in the inner ear of the African cichlid fish *Astatotilapia burtoni*, a species that is endemic to shallow shore pools of Lake Tanganyika. *A. burtoni *live in a lek-like social system where males exist in one of two phenotypes: 1) dominant territorial males (~10-30% of population) that are brightly colored, aggressively defend a spawning territory, and actively court and spawn with females; and 2) subordinate non-territorial males that school with and resemble females in coloration, perform subordinate behaviors, and do not court females [26]. Males can rapidly and reversibly switch between dominant and subordinate appearance and behavior depending solely on the composition of the social environment. Importantly, this social transformation in males causes a suite of behavioral and physiological changes along the reproductive axis [27]. Females do not have a similarly organized social system, but typically school with subordinate males and enter the territories of dominant males only to eat and spawn. After spawning, females rear the developing young in their mouths (mouth brooding) for 2 weeks before releasing them, and then physiologically recover before spawning again. While visual cues are critically important for social behaviors in this species, hydrodynamic and acoustic signals detected by the lateral line and inner ear are also likely essential for perception of social information. Territorial male *A. burtoni *produce courtship sounds, which are a series of pulses emitted during quivering behaviors in the presence of a female (Figure 1 and Maruska and Fernald, personal observations), as well as hydrodynamic sounds associated with body movements during specific behavioral acts [26,28]. These *A. burtoni *courtship sounds are very similar to those described in other cichlids [29-31] and may provide crucial information on reproductive condition, dominance status, fish size, or location of feeding and spawning territories that complement visual cues. Since auditory cues may be important for social behaviors in this species, the inner ear could be an important substrate for steroid-mediated auditory and social plasticity.

Our goal for this study was to test whether steroid receptor mRNA expression in the main hearing organ of a teleost fish, the saccule, varied between sexes or across reproductive and social states within a sex, and whether these changes were correlated with circulating steroid levels. To our knowledge, this is the first comprehensive study of sex-, social-, and reproductive-related steroid receptor mRNA expression in the peripheral auditory system of any vertebrate. Our data provide support for the hypothesized conserved action of steroid hormones in the vertebrate inner ear.

## Results

### GSI and circulating steroid levels

**Figure 2:**
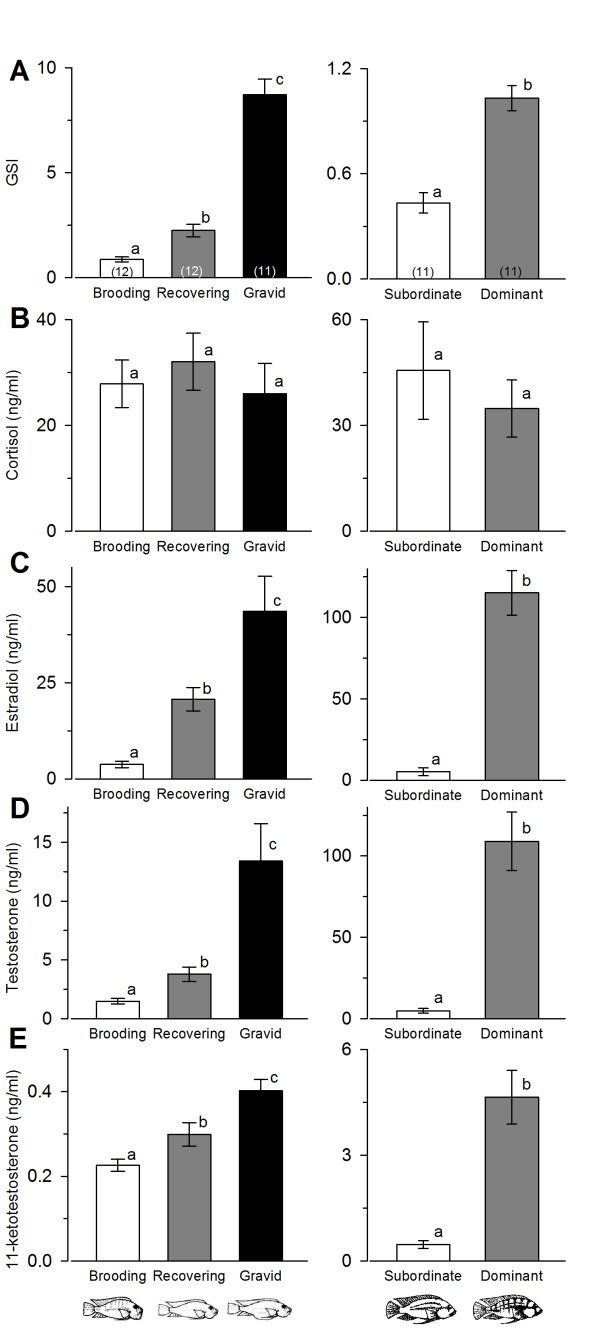
**Gonadosomatic index and circulating steroid hormone levels in male and female *Astatotilapia burtoni***. (A) Gonadosomatic index (GSI) differed among brooding, recovering, and gravid females, and between dominant territorial males and subordinate non-territorial males. (B) Circulating cortisol levels did not differ among females, or between male phenotypes. (C) Serum estradiol (E_2_) levels differed among females in all three reproductive states, and dominant males had higher E_2 _levels compared to subordinate males. (D) Circulating testosterone levels also differed among brooding, recovering, and gravid females, and were approximately 10-fold higher in dominant compared to subordinate males. (E) Serum 11-ketotestosterone levels differed among all three female groups, and dominant males had 10-fold higher levels than subordinate males. Data are plotted as mean ± SE. Bars with different letters within a sex represent significant differences (*p *< 0.05) and sample sizes are indicated within each bar.

GSI differed among all three reproductive stages in females (KW, H = 28.10, *p *< 0.001; Dunn's test, *p *< 0.05), where gravid females had mean values about four-fold greater than recovering females and ten-fold greater than brooding females (Figure 2A). GSI was also two-fold greater in dominant males compared to subordinate males (Student's t-test, t = -6.50, *p *< 0.001) (Figure 2A).

Circulating cortisol levels did not differ significantly among females in different reproductive states (KW ANOVA, H = 3.52, *p *= 0.172) nor between male phenotypes (Mann-Whitney, U = 51.5, *p *= 0.833) (Figure 2B). There was no correlation between serum cortisol levels and GSI for either males (*r *= 0.003, *p *= 0.990) or females (*r *= 0.121, *p *= 0.490).

Serum E_2 _levels differed among all three female reproductive phases where gravid individuals had concentrations two-fold higher than recovering and over 10-fold higher than brooding individuals (ANOVA, F = 14.09, *p *< 0.001) (Figure 2C). In males, dominant individuals had serum E_2 _levels over 20-times higher than subordinate individuals (Mann-Whitney, U = 0.0, *p *< 0.001). GSI was also positively correlated with circulating E_2 _levels in both females (*r *= 0.49, *p *= 0.003) and males (*r *= 0.78, *p *< 0.001).

Circulating T and 11-KT levels also differed among all three female reproductive phases in the same pattern as E_2 _(T: ANOVA, H = 22.73, *p *< 0.001, Dunn's test, *p *< 0.05; 11-KT: ANOVA, F = 14.14, *p *< 0.001, Tukey's test, *p *< 0.05) (Figures 2D, E). Dominant males had approximately 10-fold higher serum T (Mann-Whitney, U = 1.0, *p *< 0.001) and 11-KT (Mann-Whitney, U = 1.0, *p *< 0.001) levels compared to subordinate males (Figures 2D, E). GSI was also positively correlated with serum T (females: *r *= 0.51, *p *< 0.001; males: *r *= 0.49, *p *= 0.025) and 11-KT (females: *r *= 0.67, *p *< 0.001; males: *r *= 0.49, *p *< 0.001) concentrations in both females and males.

### Androgen receptor mRNA expression in the saccule

**Figure 3:**
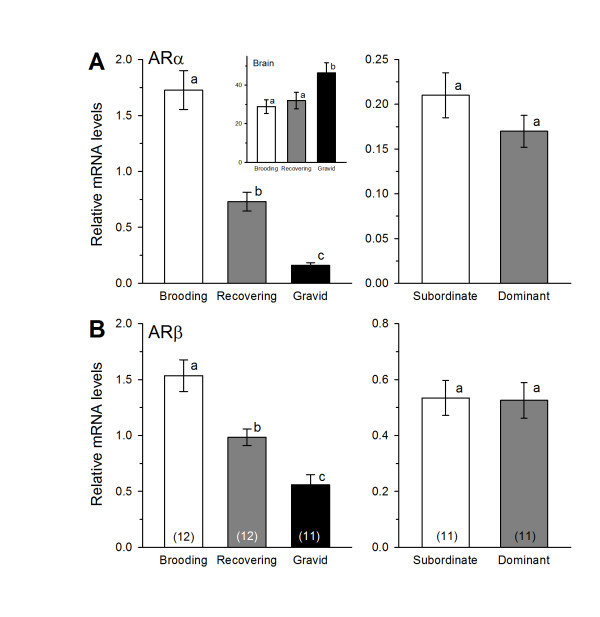
**Androgen receptor mRNA expression in the saccule of male and female *Astatotilapia burtoni***. (A) mRNA levels of ARα differed among brooding, recovering and gravid females, but not between dominant and subordinate males. *Inset*: ARα mRNA levels in the brain were higher in gravid females compared to both recovering and brooding animals. (B) mRNA levels of ARβ also differed among all three female reproductive states, but did not differ among subordinate and dominant males. Data are plotted as relative mRNA levels (mean ± SE) referenced to the geometric mean of two housekeeping genes (18s and G3PDH). Bars with different letters represent significant differences (*p *< 0.05) and sample sizes are indicated within each bar on the bottom graphs.

ARα and ARβ mRNA levels differed among all three female reproductive phases where brooding individuals had the highest levels followed by recovering and then gravid animals (ARα: KW, H = 25.75, *p *< 0.001, Dunn's test, *p *< 0.05; ARβ: 1-way ANOVA, F = 18.58, *p *< 0.001, Tukey's test, *p *< 0.05) (Figures 3A, B). In contrast, there was no difference in either ARα or ARβ mRNA levels of subordinate compared to dominant males (Student's t-tests, ARα: t = 1.30, *p *= 0.207; ARβ: t = 0.098, *p *= 0.923) (Figures 3A, B).

### Estrogen receptor and aromatase mRNA expression in the saccule

**Figure 4:**
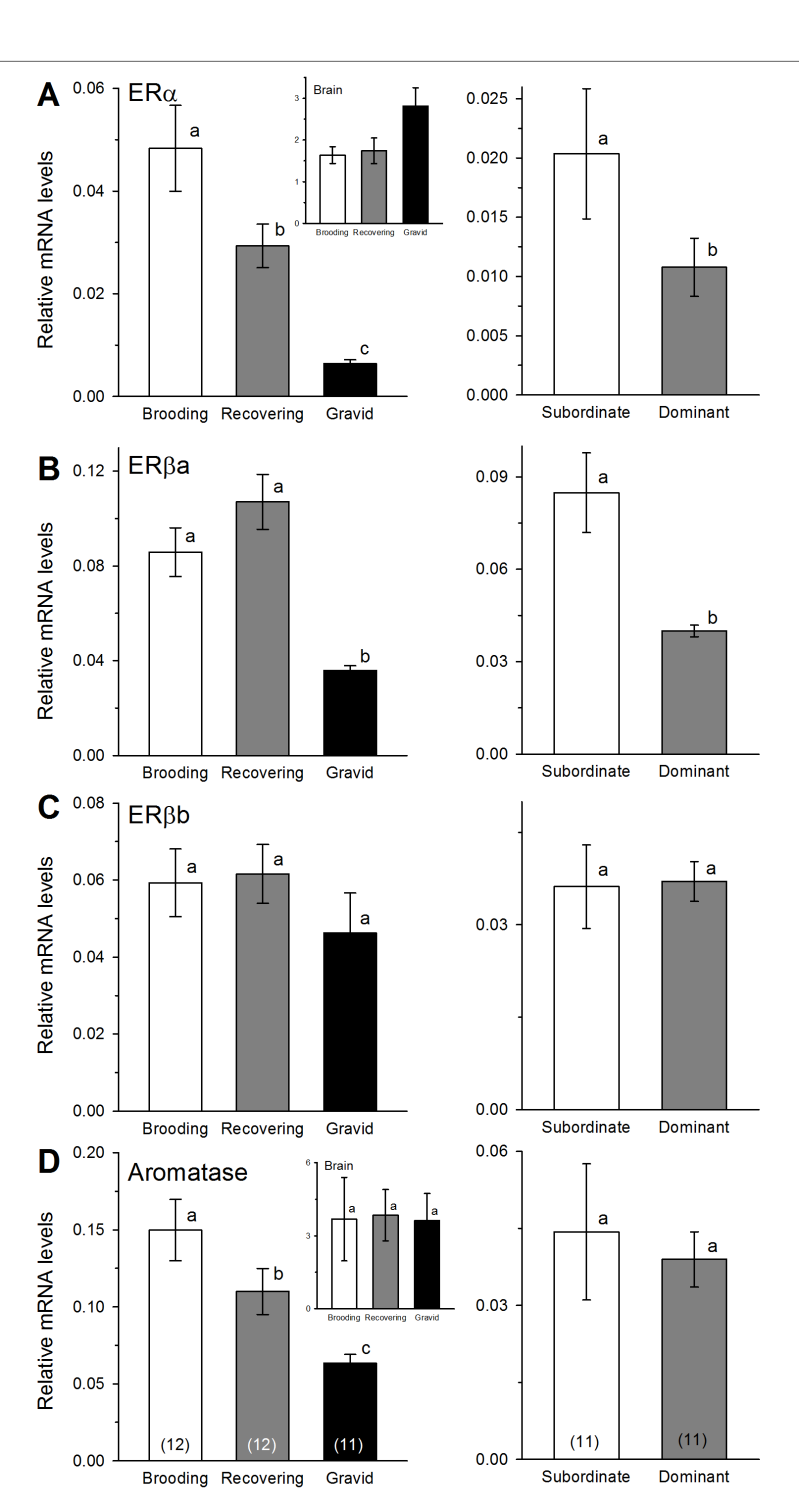
**Estrogen receptor and aromatase mRNA expression in the saccule of male and female *Astatotilapia burtoni***. (A) ERα mRNA levels differed among all three reproductive phases in females, and subordinate males had higher ERα levels compared to dominant males. *Inset*: Gravid females had greater ERα mRNA levels in the brain compared to brooding and recovering animals. (B) ERβa mRNA levels were lower in gravid females compared to both brooding and recovering animals, and subordinate males had higher levels than dominant males. (C) There was no difference in saccular ERβb mRNA expression in females or males. (D) Aromatase (CYP19a) mRNA levels differed among female reproductive phases, but there was no difference between male phenotypes. *Inset*: There was no difference in brain aromatase mRNA levels among different female groups. Data are plotted as relative mRNA levels (mean ± SE) referenced to the geometric mean of two housekeeping genes (18s and G3PDH). Bars with different letters represent significant differences (*p *< 0.05) and sample sizes are indicated within each bar on the bottom graphs.

In females, ERα mRNA levels differed among all three reproductive phases with brooding individuals showing the highest levels, followed by recovering and then gravid animals (KW, H = 18.0, *p *< 0.001, Dunn's test, *p *< 0.05) (Figure 4A). In males, subordinates had greater levels of ERα compared to dominant animals (Mann-Whitney rank sum test, U = 27.0, *p *= 0.030) (Figure 4A).

ERβa mRNA levels were lower in gravid females compared to both brooding and recovering individuals (KW, H = 16.29, *p *< 0.001, Dunn's test, *p *< 0.05) (Figure 4B). Subordinate males also had two-fold higher ERβa levels compared to dominant males (Mann-Whitney rank sum test, U = 16.0, *p *= 0.004) (Figure 4B). ERβb mRNA levels did not differ among reproductive phases in females (KW, H = 5.63, *p *= 0.060), nor between social states in males (Student's t-test, t = -0.112, *p *= 0.912) (Figure 4C).

Aromatase expression in the saccule of females differed among all three reproductive phases in an identical pattern to that of ERα (1-way ANOVA, F = 8.04, *p *= 0.001, Tukey's test, *p *< 0.05) (Figure 4D). In males, there was no difference in mRNA levels of aromatase between subordinate and dominant phenotypes (Mann-Whitney rank sum test, U = 51.0, *p *= 0.555) (Figure 4D).

### Corticosteroid receptor mRNA expression in the saccule

**Figure 5:**
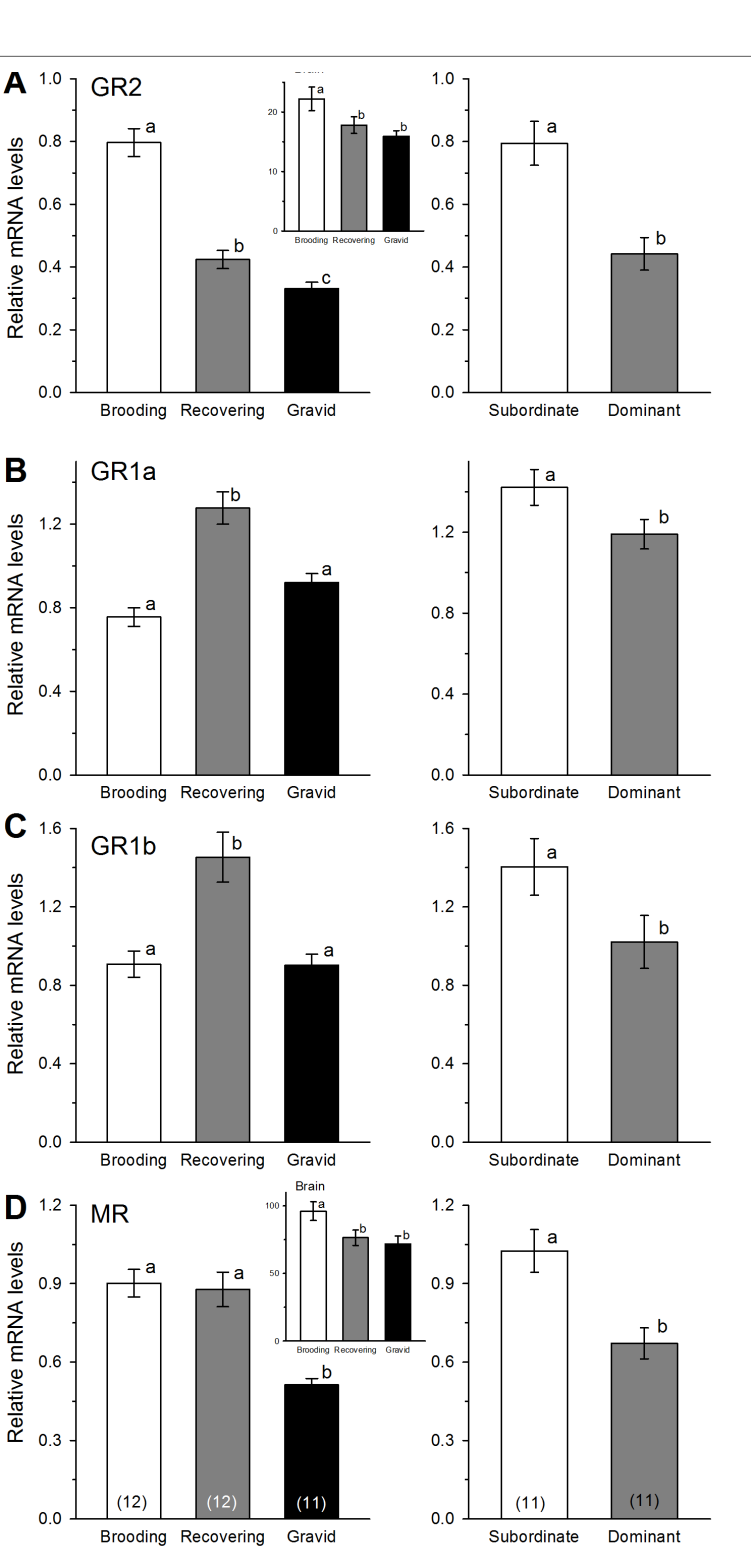
**Corticosteroid receptor mRNA expression in the saccule of male and female *Astatotilapia burtoni***. (A) GR2 mRNA levels differed among all three female groups, and subordinate males had higher mRNA levels compared to dominant males. *Inset*: Brooding females had higher GR2 mRNA levels in the brain compared to both recovering and gravid animals. (B) GR1a mRNA levels were higher in recovering females compared to brooding and gravid individuals, and subordinate males had higher GR1a mRNA levels than dominant males. (C) Similar to GR1a, mRNA levels of GR1b were higher in recovering females compared to brooding and gravid individuals, and subordinate males had higher GR1b mRNA levels than dominant males. (D) MR mRNA levels were lower in gravid females compared to both brooding and recovering animals, and subordinate males had higher MR mRNA levels compared to dominant males. *Inset*: Brooding females had higher brain MR mRNA levels compared to both recovering and gravid animals. Data are plotted as relative mRNA levels (mean ± SE) referenced to the geometric mean of two housekeeping genes (18s and G3PDH). Bars with different letters represent significant differences (*p *< 0.05) and sample sizes are indicated within each bar on the bottom graphs.

GR2 mRNA levels differed among the three female reproductive phases with the highest levels found in brooding females, followed by recovering and gravid individuals (1-way ANOVA, F = 54.76, *p *< 0.001, Tukey's test, *p *< 0.05) (Figure 5A). GR1a and GR1b mRNA levels were higher in recovering females compared to both brooding and gravid animals (1-way ANOVA, GR1a: F = 21.48, *p *< 0.001, Tukey's test, *p *< 0.05; GR1b: F = 12.28, *p *< 0.001, Tukey's test, *p *< 0.05) (Figures 5B, C). MR levels were similar in brooding and recovering females, but both were greater than levels found in gravid females (KW, H = 16.99, *p *< 0.001, Dunn's test, *p *< 0.05) (Figure 5D). In males, subordinate animals had higher levels of all four CRs (GR2, GR1a, GR1b, MR) compared to dominant animals (Student's t-tests GR2: t = 4.04, *p *< 0.001; GR1a: t = 2.02, *p *= 0.050; GR1b: t = 1.95, *p *= 0.050; Mann-Whitney rank sum test MR: U = 17.0, *p *= 0.005) (Figures 5A-D).

### Steroid receptor mRNA expression in the female brain

The pattern of steroid receptor mRNA levels in the female brain differed from that observed in the inner ear. Brain ARα mRNA levels were greater in gravid females compared to both brooding and recovering females (KW, H = 7.01, *p *= 0.030, Dunn's test, *p *< 0.05) (Figure 3A inset). Gravid females also showed higher levels of ERα than both brooding and recovering females (1-way ANOVA, F = 3.67, *p *= 0.037, Tukey's test, *p *< 0.05) (Figure 4A inset). In contrast to the saccule, there was no difference among females in aromatase expression in the brain (KW, H = 0.742, *p *= 0.690) (Figure 4D inset). GR2 and MR mRNA levels were higher in brooding females compared to both gravid and recovering females (GR2: 1-way ANOVA, F = 4.37, *p *= 0.021, Tukey's test, *p *< 0.05; MR: 1-way ANOVA, F = 4.27, *p *= 0.023, Tukey's test, *p *< 0.05) (Figures 5A, 5D insets).

### Sex differences in saccular steroid receptor mRNA levels

On average, females had two to five-fold greater mRNA levels of both ARs compared to males (Mann-Whitney rank sum tests, *p *< 0.05) (Table 1). Females also had higher levels of aromatase and ERα, but not ERβa or ERβb, compared to males (Mann-Whitney rank sum tests, *p *< 0.05) (Table 1). In contrast, mRNA levels of all four CRs in the saccule were higher in males compared to females (Mann-Whitney rank sum tests, *p *< 0.05) (Table 1).

**Table 1:** Sex comparisons of steroid receptor and aromatase mRNA expression in the saccule of *Astatotilapia burtoni*.

Receptor subtype	U statistic	*p*	Summary
ARα	135.0	**<0.001**	**F > M**

ARβ	224.0	**0.009**	**F > M**

ERα	258.0	**0.050**	**F > M**

ERβa	365.0	0.749	F = M

ERβb	333.0	0.399	F = M

Aromatase	106.0	**<0.001**	**F > M**

GR2	190.0	**0.001**	**F < M**

GR1a	31.0	**<0.001**	**F < M**

GR1b	263.0	**0.045**	**F < M**

MR	174.0	**<0.001**	**F < M**

Female data were pooled from brooding, recovering and gravid individuals, while male data were pooled from subordinate and dominant individuals. U statistics and *p*-values are from Mann-Whitney Rank Sum tests and bold values indicate differences at *p *< 0.05 (Bonferroni correction *p *< 0.005)

### Correlations between receptor mRNA levels and circulating steroid levels and GSI

Correlations between steroid receptor mRNA levels in the saccule and circulating CS, E_2_, T, and 11-KT levels are summarized in Table 2 (females) and Table 3 (males). There was a positive correlation between serum CS levels and GR1a expression in the saccule of males, but no other relationships between any GR type and CS levels were found in either sex.

**Table 2:** Correlations between steroid receptor and aromatase mRNA levels in the saccule and circulating steroid hormone concentrations in female *Astatotilapia burtoni*.

*Females*	CS		E_2_		T		11-KT	
	***r***	***p***	***r***	***p***	***r***	***p***	***r***	***p***
	
ARα	-0.12	0.495	-0.60	**<0.001**	-0.49	**0.003**	-0.51	**0.002**

ARβ	0.001	0.995	-0.50	**0.002**	-0.40	**0.018**	-0.46	**0.006**

ERα	-0.10	0.560	-0.47	**0.004**	-0.40	**0.018**	-0.43	**0.010**

ERβa	0.03	0.869	-0.36	**0.032**	-0.40	**0.018**	-0.34	**0.046**

ERβb	-0.04	0.822	-0.23	0.179	-0.15	0.394	-0.21	0.233

Aromatase	-0.03	0.853	-0.39	**0.019**	-0.36	**0.033**	-0.40	**0.017**

GR2	-0.18	0.291	-0.54	**<0.001**	-0.43	**0.009**	-0.49	**0.003**

GR1a	0.29	0.098	0.20	0.260	0.08	0.653	0.24	0.174

GR1b	0.13	0.457	-0.01	0.935	-0.16	0.350	-0.01	0.962

MR	0.11	0.513	-0.43	**0.010**	-0.41	**0.014**	-0.33	**0.052**

CS, cortisol; E_2_, estradiol; T, testosterone; 11-KT, 11-ketotestosterone. Correlation coefficients (*r*) and *p*-values are from Pearson Product moment or Spearman rank tests. P-values in bold indicate significant relationships at *p *< 0.05 (Bonferroni correction *p *< 0.0013).

**Table 3:** Correlations between steroid receptor and aromatase mRNA levels in the saccule and circulating steroid hormone concentrations in male *Astatotilapia burtoni*.

*Males*	CS		E_2_		T		11-KT	
	***r***	***p***	***r***	***p***	***r***	***p***	***r***	***p***
	
ARα	0.01	0.975	-0.21	0.351	-0.53	**0.013**	-0.56	**0.008**

ARβ	-0.11	0.621	0.14	0.560	-0.29	0.194	-0.46	**0.034**

ERα	0.54	**0.012**	-0.46	**0.010**	-0.23	0.324	-0.10	0.654

ERβa	0.30	0.183	-0.56	**0.008**	-0.54	**0.012**	-0.54	**0.012**

ERβb	0.27	0.235	0.09	0.708	-0.04	0.865	-0.09	0.694

Aromatase	0.32	0.151	-0.06	0.797	-0.20	0.387	-0.16	0.496

GR2	0.30	0.184	-0.69	**<0.001**	-0.50	**0.023**	-0.35	0.121

GR1a	0.45	**0.040**	-0.53	**0.015**	-0.27	0.232	-0.09	0.669

GR1b	0.21	0.365	-0.63	**0.002**	-0.40	0.072	-0.22	0.344

MR	0.11	0.636	-0.64	**0.002**	-0.47	**0.032**	-0.34	0.128

CS, cortisol; E_2_, estradiol; T, testosterone; 11-KT, 11-ketotestosterone. Correlation coefficients (*r*) and *p*-values are from Pearson Product moment or Spearman rank tests. P-values in bold indicate significant relationships at *p *< 0.05 (Bonferroni correction *p *< 0.0013).

There was a negative correlation between serum E_2 _levels and both ERα and ERβa in the saccule of both males and females, but no relationship between E_2 _and ERβb in either sex (Tables 2, 3). Further, circulating E_2 _levels were also negatively correlated with aromatase expression in the saccule of females but not males.

There was a negative correlation between circulating androgen (T and 11-KT) levels and ARα, ARβ, ERα, ERβa and aromatase in the saccule of females (Table 2). In males, there was a negative correlation between circulating T levels and saccular expression of ERβa and ARα, but not ARβ (Table 3). Circulating T levels were also positively correlated with serum E_2 _levels in both females (*r *= 0.87, *p *< 0.001) and males (*r *= 0.67, *p *< 0.001).

In contrast to the saccule, receptor mRNA levels in the female whole brain samples were not well correlated with circulating steroid levels, with the exception of a positive correlation between ARα and T (*r *= 0.36, *p *= 0.034). Serum CS was not correlated with brain mRNA levels of GR2 (*r *= 0.16, *p *= 0.376) or MR (*r *= 0.12, *p *= 0.504). Brain ERα and aromatase mRNA levels were also not correlated with either E_2 _(ERα: *r *= 0.24, *p *= 0.181; aromatase: *r *= 0.08, *p *= 0.667) or T (ERα: *r *= 0.25, *p *= 0.167; aromatase: *r *= -0.07, *p *= 0.680).

Correlations between steroid receptor mRNA levels in the saccule and GSI are summarized in Table 4. In females, GSI was negatively correlated with mRNA levels of all sex-steroid receptor subtypes except ERβb, as well as negatively correlated with the corticosteroid receptors GR2 and MR. In males, there was only a single negative correlation between GSI and GR1a.

**Table 4:** Correlations between steroid receptor and aromatase mRNA levels in the saccule and gonadosomatic index for male and female *Astatotilapia burtoni*.

Receptor subtype	Females		Males	
	***r***	***p***	***r***	***p***
	
ARα	-0.67	**< 0.001**	0.40	0.122

ARβ	-0.57	**< 0.001**	0.24	0.374

ERα	-0.56	**< 0.001**	-0.36	0.173

ERβa	-0.56	**< 0.001**	-0.19	0.479

ERβb	-0.16	0.358	-0.18	0.496

Aromatase	-0.49	**0.003**	-0.26	0.327

GR2	-0.60	**< 0.001**	-0.47	0.070

GR1a	0.002	0.992	-0.62	**0.011**

GR1b	-0.13	0.475	-0.24	0.380

MR	-0.65	**< 0.001**	-0.47	0.068

Correlation coefficients (*r*) and *p*-values are from Pearson Product moment or Spearman rank tests. P-values in bold indicate significant relationships at *p *< 0.05 (within sex Bonferroni correction *p *< 0.005).

## Discussion

Our data on mRNA expression patterns of steroid receptors in the saccule of *A. burtoni *demonstrate that 1) multiple steroid receptor subtypes are expressed in the inner ear of a single fish species; and 2) steroid receptor mRNA levels vary with sex, social status in males, and reproductive condition in females. While previous studies in diverse taxa show the presence of some steroid receptors in both sensory and non-sensory tissues of the inner ear, their expression patterns are rarely quantified. Our study confirms expression of multiple ERs, ARs, CRs, and aromatase in the inner ear of a single vertebrate species, and reveals a previously undescribed plasticity in receptor transcript abundance related to both internal reproductive state and the external social environment. These data provide support for the hypothesis that, in addition to circulating steroid levels, auditory function and inner ear homeostasis could also be regulated by variations in receptor levels in the ear. Future studies are needed, however, to determine whether or not these changes reflect functional significance.

To our knowledge, this is the first study to demonstrate the presence of ERβ, and multiple ARs, GRs and MR mRNA in the inner ear of any non-mammalian vertebrate (but see [22]). Our data indicate that steroid receptor expression, and therefore steroid sensitivity, in peripheral auditory structures may be a conserved feature among vertebrates. However, a whole-mount *in situ *hybridization study in zebrafish *Danio rerio *did not detect ER mRNA within the inner ear, but did show abundant ER expression within the morphologically and functionally similar lateral line neuromasts [32], which also raises the possibility of species-specific differences even within broad taxonomic groups. Unique distribution profiles of multiple steroid receptor isoforms in the same species is common among vertebrates and suggests sub-functionalization [24,33]. In teleost fishes, little is known about ligand-induced activation profiles of receptor subtypes in a single species, but there is evidence for both differential activation of ARs, CRs, and ERs by the same ligand, as well as activation of a single receptor subtype by multiple ligands [34-38]. This pattern of multiple interactions highlights the potential complexity of steroid-mediated transduction pathways, and given the profound organizational and activational effects of steroids in vertebrates, implicates the auditory system as an important target for all classes of steroid hormones.

### Corticosteroid receptor mRNA expression

The corticosteroid signaling system in teleost fishes is complex, and in *A. burtoni*, all four types of CRs are activated by both cortisol and aldosterone (although it is thought that teleosts do not synthesize aldosterone-like compounds) [35]. Expression levels of each CR subtype also differ among body tissues, which is indicative of functional specialization [35]. Here we show further differential expression of each CR subtype within the inner ear that is related to reproductive state in females and social status in males. However, there was little evidence that changes in CR expression in the ear are regulated by circulating cortisol levels, with the exception of the positive correlation between GR1a and serum CS levels in males. It is possible that CR expression is regulated by local glucocorticoid levels within the fluid of the inner ear that differs from circulating cortisol measures, or that CRs are regulated independently of their ligands. Alternatively, CRs may be regulated by compounds in the corticosteroid biosynthetic pathway other than CS such as 11-deoxycorticosterone or some yet unidentified aldosterone-like hormone. Glucocorticoids can both up-regulate and down-regulate synthesis of specific proteins within cochlear tissue of the rat [39], which highlights the importance of examining CR subtype distribution, abundance, and transduction pathways. In mammals, serum mineralocorticoid and glucocorticoid levels are correlated with Na^+^, K^+^-ATPase activity (an enzyme thought to play an important role in endolymph production) in the cochlea [40,41], but the hypothesis that Na^+^, K^+^-ATPase levels are regulated by GR or MR expression could not be verified [42,43]. Nevertheless, corticosteroids and CR expression are likely important for maintenance of ion balance and metabolic state of the inner ear across vertebrates, but the mechanisms remain to be elucidated.

### Estrogen receptor and aromatase mRNA expression

Saccular levels of ERα, ERβa and aromatase were lower in female *A. burtoni *undergoing ovarian recrudescence in preparation for spawning compared to mouth brooding females that put little investment into egg growth until the parental care period is over. However, it is important to note that changes in the CYP19b aromatase form may be different from the CYP19a form measured here, and requires future investigation. Serum E_2 _levels in females were also negatively correlated with ERα, ERβa, and aromatase mRNA levels in the saccule, indicating down-regulation of E_2_-sensitive pathways in the inner ear during ovarian recrudescence associated with high E_2 _production. High circulating estrogen levels during pregnancy are also associated with a down-regulation of ERs in the inner ear of mother rats [8]. Unfortunately, the relationship between steroid receptor expression in the ear, circulating hormone levels, and hearing ability within a single species are largely unknown so it is difficult to comment on whether high estrogens and low ER expression translate into changes in hearing. For example, in the midshipman fish, gravid females alter their auditory frequency tuning so they can better hear the male's advertisement call during the breeding season, a phenomenon mimicked by exogenous treatment with E_2 _or T [3,44]. While these females have high circulating E_2 _levels just prior to the spawning period (i.e., pre-nesting) [45], and they do express ERα and CYP19b aromatase in the saccule [10], it is not known whether this seasonal plasticity is regulated by changes in ER expression in the ear. In mice however, aromatase knockouts (with little to no measurable serum E_2 _levels) show decreased ERβ staining in the cochlea, and greater threshold shifts in response to acoustic trauma compared to wild-type individuals, but no difference in normal hearing thresholds [46]. This indicates a very specific protective effect of ERβ in response to trauma, which may also be species-specific. In *A. burtoni*, females can reproduce and brood young year-round and changes in inner ear steroid receptor expression are coupled to gonadal state and serum hormone levels, which suggests some potential yet unknown consequence on saccular maintenance or function.

In contrast to ERα and ERβa, ERβb mRNA levels did not differ among females of different reproductive states or between males of different social status. ERβb mRNA levels in the ear were also not correlated with circulating E_2 _levels in either sex, which suggests this subtype probably does not serve as a substrate for feedback regulation from estrogens. In zebrafish, direct E_2 _exposure resulted in strong stimulation of ERα expression, but reduced ERβ1 (equivalent to ERβb of *A. burtoni*) and had little effect on ERβ2 (equivalent to ERβa of *A. burtoni*) mRNA in the liver [34], indicating that ER subtypes are differentially regulated by the same ligand and likely serve distinct physiological functions. In mammals, the ERβ subtype is important for maintaining hearing capabilities by preventing age-related hearing loss, and providing protection from acoustic trauma via a mechanism that involves brain-derived neurotrophic factor (BDNF) [7,46] and possibly activation of intermediate filaments [47]. The absence of status- and reproductive-related variations in ERβb in *A. burtoni *suggests that this subtype may be important for regular maintenance of auditory function or homeostasis in non-mammalian vertebrates as well.

### Androgen receptor mRNA expression

Androgen receptors in the saccule of female *A. burtoni *differed among reproductive states, and females had two to three-fold greater AR mRNA levels than males. AR mRNA expression in the inner ear has only been demonstrated in one other vertebrate, the midshipman fish [11,22], so it is difficult to evaluate whether these reproductive- and sex-related variations seen in *A. burtoni *are present in other taxa. AR expression in the ear, however, was negatively correlated with circulating androgens in females suggesting down-regulation of ARs by high androgen levels produced during oocyte growth and maturation. This ligand-receptor regulatory mechanism may also explain the elevated AR levels in the ear of females that have lower androgen levels compared to males. In contrast to the well-known effects of estrogens on auditory function, relatively little is known about how androgens may influence hearing or inner ear homeostasis. Prenatal exposure to high levels of androgens are hypothesized to masculinize the cochlea and contribute to sex-differences in click-induced otoacoustic emissions in mammals [48], but since ARs have not yet been found in the cochlea, it is not known whether these organizational effects are mediated directly by ARs or possibly via ERs after aromatization. In the midshipman fish, testosterone causes a shift in frequency tuning of saccular afferents that is similar to estradiol treatment [3], and coupled with the recent finding of AR in the ear of that species [11] suggests direct androgen influence at the periphery. Our discovery of multiple ARs in the adult vertebrate inner ear and their plastic expression further supports the hypothesis that androgens can also have direct activational effects on the ear, but the mechanisms and impact on auditory function require further study.

### Comparison of steroid receptor mRNA expression between the saccule and brain

The patterns of steroid receptor gene expression in the inner ear of *A. burtoni *differed from that observed in whole brain samples from the same female individuals. For example, gravid females with high plasma androgen levels had the lowest levels of AR in the saccule, but had the highest AR levels in the brain. This pattern was also true for males where sex-steroid receptor levels in the ear were higher in subordinate compared to dominant animals, but levels in sub-regions of the brain were higher in dominant compared to subordinate males [49]. Further, there were fewer correlations between circulating steroid levels and receptor mRNA levels in the brain than there were in the inner ear. These data raise the possibility that steroid receptors in the brain and inner ear are regulated by different mechanisms. For example, receptor levels in the ear may be more influenced by peripheral steroid levels, while the brain may depend less on circulating hormones and more on local steroid production within specific brain regions to regulate receptors (i.e., neurosteroids) [50]. It is also important to note that steroid receptors are found in many different brain nuclei and measures of receptor expression in whole brains, or large heterogeneous brain regions, may be very different from localized changes in specific processing centers.

### Functional consequences

In the cichlid fish *A. burtoni*, social status regulates reproductive opportunity and fertility. Subordinate males have a suppressed brain-pituitary-gonad axis with small GnRH1 neurons, low levels of gonadotropin hormones, reduced testicular size, and low circulating levels of sex steroids compared to dominant reproductively active males [27,51-53]. Here we provide evidence that the peripheral auditory system is also a substrate for social regulation of gene expression. Levels of ERα, ERβa, and all four CRs were higher in the inner ear of subordinate compared to dominant males. However, the negative correlations between circulating sex-steroid levels and receptor expression in the ear indicates that it may not be social status *per se *that regulates this plasticity, but rather, transformation of the reproductive axis causes variations in serum hormone concentrations that in turn regulate receptor levels. While this mechanism may explain the pattern of higher ER levels in the saccule of subordinate males, it does not rationalize the fact that levels of both ARs are equivalent among male phenotypes despite subordinate animals having much lower serum androgen concentrations. Thus it is possible that social cues do have direct effects on AR expression in the inner ear of males that is independent of ligand-mediated control from the general circulation. These data also suggest that the regulatory mechanisms of receptor expression in the ear may differ between the sexes such that receptor expression in females is influenced by internal physiological state, while in males, external social cues coupled with yet unknown pathways may play a larger role.

Subordinate *A. burtoni *males also have higher CR expression in the saccule, a difference which for subtypes other than GR1a is not explained by either GSI or circulating CS levels. Social suppression alone may modulate inner ear CR expression directly, or act via mechanisms independent of the brain-pituitary-gonad or hypothalamic-pituitary-interrenal axis. In previous studies, serum CS levels often do not differ between subordinate and dominant *A. burtoni *males when examined at ≤ 5 weeks in an established community, but subordinate males do have much higher CS levels after longer time periods (~7 weeks) [54,55]. Thus it is possible that circulating CS levels would better correlate with CR expression at later time points. Examination of steroid receptor levels and serum steroid levels of males transitioning between subordinate and dominant states, or manipulation of plasma steroid levels independent of social status, will help resolve whether social status or reproductive state-related hormone concentrations regulate receptor expression in the ear.

Estradiol appears to improve hearing ability in all vertebrate models studied to date [12], particularly in females. In *A. burtoni*, gravid females have high circulating E_2 _levels, and were previously shown to prefer dominant over subordinate males, as well as smaller more active males over less active ones [56]. In cichlids, the peak frequency of courtship sounds is related to fish body size [29,30], thus providing females with an honest signal for mate choice decisions. For example, more active small males would produce more frequent courtship sounds of higher frequency, providing gravid females with auditory information to supplement visual and other sensory cues that can be used to choose a mate. Interestingly, in another teleost fish, the midshipman, estradiol shifts auditory tuning in females towards higher frequencies as an adaptation for coupling of sender-receiver physiology [3]. Our finding of reproductive-related plasticity in steroid receptor expression in the ear of females raises the possibility that hormones may profoundly influence both female mate choice and the evolution of male signaling behaviors for acoustic communication. Future studies are needed, however, to determine the relative importance of auditory cues during courtship, and the effects of estradiol and other steroids on hearing ability in this and other species.

## Conclusions

Our data show the presence of multiple sex- and stress-related steroid receptors in the fish inner ear, and that their mRNA levels vary with sex, internal physiological state (i.e., circulating hormones) and social status. This is the most comprehensive study of sex-, social-, and reproductive-related steroid receptor mRNA expression in the peripheral auditory system of any single vertebrate, and provides support for the hypothesized conserved function(s) of steroid receptor-mediated influence within the inner ear. The next steps are to discover which cell types within the fish inner ear express different steroid receptor subtypes, whether observed changes in mRNA expression are reflected in protein abundance, and what functional consequences these variations in receptor levels might have on auditory function and inner ear homeostasis.

## Methods

### Animals

Laboratory-bred adult male and female cichlid fish *Astatotilapia burtoni*, derived from wild-caught stock in Lake Tanganyika, Africa, were maintained in aquaria under environmental conditions that mimic their natural equatorial habitat (28°C; pH 8.0; 12 h light: 12 h dark with full spectrum illumination; constant aeration), and fed cichlid pellets and flakes (AquaDine, Healdsburg, CA, USA) each morning. Aquaria contained gravel-covered bottoms with terra cotta pots cut in half to serve as spawning territories. All experimental procedures were approved by the Stanford Administrative Panel for Laboratory Animal Care.

Stable dominant and subordinate males were established by initially placing two dominant territorial males from separate community tanks together in an aquarium with a single terra cotta pot territory and 4 females. In this situation, social interactions between the two males result in one male becoming dominant over the other, usually in less than an hour. The dominant male then defends the territory and uses it to court and spawn with females, while the subordinate male becomes drab-colored, is frequently chased by the newly dominant individual, and becomes reproductively suppressed. Fish were observed daily to verify that the dominant and subordinate males were stable phenotypes and maintained their social status for 4-5 weeks, a time sufficient to ensure behavioral and reproductive suppression in the subordinate animals.

Female *A. burtoni *breed year round and provide sole parental care to developing young which they brood in their mouths. Females have three distinct reproductive phases, which were selected for analysis: 1) Mouth brooding females had mouths filled with large full-term embryos (standard length 8.2 ± 1.5 mm SD) that they had been brooding for 14 days. Mouth brooding females generally do not eat and provide sole care for the developing young; 2) Gravid females had visibly swollen abdomens, numerous large "ready-to-spawn" eggs (some eggs were readily released from the ovary upon dissection in all individuals indicating they were at or near ovulation), and a correspondingly high gonadosomatic index, and; 3) Recovering females (neither gravid nor mouth brooding) were created by releasing full-term fry from mouth brooders to initiate ovarian recrudescence, and then returning them to their community tanks to recover for 12 days (~equivalent to approximately half of the average ovarian cycle period of 25-30 days) prior to sacrifice.

### Tissue preparation

All fish used in this study were sacrificed at the same time of day (9:30-10:30 am) to control for any potential diurnal changes in gene expression, and were size-matched to account for any differences due to body size (female SL, ANOVA *p *= 0.078; male SL, t-test *p *= 0.086). Fish were captured from their tanks, anesthetized in ice-cold tank water, and standard length (SL) and total body mass (BM) measured. Immediately before sacrifice by rapid cervical transection, blood samples (50-100 μl) were collected from the caudal vein by caudal severance with heparinized 100 μl capillary tubes within 2 min of capture. Blood was centrifuged for 10 min at 8000 rpm, and the plasma was removed and stored at -80°C until assayed.

The inner ear of teleosts consists of three semicircular canals (anterior, posterior, and horizontal canals) that serve a vestibular function to detect angular accelerations of the head, and the three otolithic endorgans (saccule, lagena, and utricle) that serve gravistatic and auditory functions to encode linear particle motion. The saccule is the largest otolithic endorgan in *A. burtoni *and is considered the main hearing organ in most teleost fishes. Both the left and right saccule (saccular epithelium, otolithic sac with sagitta removed, and a portion of the saccular nerve proximal to the sensory macula) were rapidly removed, flash frozen, and stored at -80°C until analysis. Testes and ovaries were also removed and weighed to calculate the gonadosomatic index [GSI = (gonad mass/body mass) × 100].

Saccular tissue was homogenized and RNA extracted following standard methods (RNeasy Micro kit, Qiagen). RNA was treated with DNase (RNase-free DNase set, Qiagen) during the isolation procedure according to kit instructions to remove contaminating genomic DNA. RNA concentration and purity was estimated from spectrophotometric absorbance (260 nm and 280 nm) for all samples. Approximately 0.25 μg of total RNA was reverse transcribed to cDNA (iscript cDNA synthesis kit, Bio-Rad) and diluted 1:5 prior to use as a template for quantitative RT-PCR reactions.

### Quantitative Reverse Transcription-PCR (qRT-PCR)

*Astatotilapia burtoni *has two androgen receptors (ARα, ARβ), three estrogen receptors (ERα, ERβa, ERβb), and four corticosteroid receptors (glucocorticoid: GR2, GR1a, GR1b; mineralocorticoid: MR) [35,49,57]. The previous nomenclature of glucocorticoid receptors in *A. burtoni *(GR1, GR2a/b) [35] was modified based on recent phylogenetic comparisons that showed the originally described *A. burtoni *GR1 was more similar to the GR2 subtype of other teleosts, and that the splice variants GR2a/b were more similar to GR1a/b [58]. Therefore, here we use the following terminology for GRs: GR2 (formerly GR1), and GR1a/b (formerly GR2a/b). Similarly, some recent reports on estrogen receptors in fishes have adopted the ESR1 (formerly ERα), ESR2a, (formerly ERβ2, ERβa, or ERγ), and ESR2b (formerly ERβ1 or ERβb) nomenclature based on official zebrafish guidelines http://zfin.org. In this manuscript, however, we refer to the originally named *A. burtoni *ER subtypes as ERα, ERβa, and ERβb to facilitate comparison with the mammalian inner ear studies that also use the ERα and ERβ terminology.

Quantitative RT-PCR was used to measure mRNA expression of the abovementioned nine different steroid receptor subtypes, plus the aromatase enzyme, from the saccule of both males and females. Whole brains (without olfactory bulbs) were also collected from all of the females sampled in this study (RNA isolated as above except that RNeasy mini kits were used and 1.0 μg RNA was reverse transcribed to cDNA) and used to measure steroid receptor levels representative of each receptor type (ARα, ERα, aromatase, GR2, MR) to test for differential regulation between the inner ear and another relevant steroid-sensitive tissue. Brains from males were not analyzed for gene expression because they were used as part of a separate study, and sex-steroid receptors in the brain of dominant and subordinate males were already measured in a previous study [49]. The iQ Sybr Green supermix (Bio-Rad) was used for qRT-PCR reactions with gene-specific primers. Aromatase primers were designed based on the previously cloned *A. burtoni *sequence (Genbank #AF114716), which is most similar to the ovarian CYP19a form: forward 5'-TTG TGG GTG AGA GAC AGC TTC AGA-3'; reverse 5'-TGT TTG TGC CCT TCG GTA TCC TGT-3' (165 bp product). Primers for all steroid receptor genes and the reference genes, 18s rRNA and glyceraldehyde 3-phosphodehydrogenase (G3PDH), were commercially synthesized and identical to those used in previous studies [35,49,59,60]. Each primer pair produced a single melting curve peak in the presence of cDNA template, and showed no amplification when water was used as a template in the reaction mix, or when reverse transcriptase was omitted from the cDNA synthesis reaction (negative controls). qRT-PCR was performed on an iCycler (MyiQ, Bio-Rad, Hercules, CA) and the reaction progress in 30 μl volumes was monitored by fluorescence detection at 490 nm during each annealing step. Reaction parameters were 3 min at 95°C followed by 45 cycles of 95°C, 60°C, and 72°C for 30 s each, and followed by a melting curve analysis over the temperature range of 95°C to 50°C (decrease by 0.5°C increments each cycle). Samples were loaded into 96-well plates such that each plate measured 1-2 genes and contained samples from each of the five experimental groups. Amplification occurred prior to cycle 30 for all genes and all samples (mean CT values for all 10 target genes ranged from 24.52 - 29.40). All reactions were performed in duplicate and several reaction products per gene were verified by DNA sequencing (Sequetech, Mountain View, CA).

Fluorescence thresholds for each sample were automatically measured (MyiQ software, Bio-Rad) and then PCR Miner [60] was used to calculate reaction efficiencies and cycle thresholds from the fluorescence readings of individual wells during the reaction. This curve-fitting real-time PCR algorithm objectively calculates reaction efficiency and the fractional cycle number at threshold (CT) of the amplification curve for more accurate computation of mRNA levels. By using the kinetics of individual reactions, estimates of efficiency and CT are independent of the specific equipment used to perform PCR reactions and data can be reliably compared across plates. The relative amount of mRNA was then normalized to the geometric mean of two housekeeping genes (18s and G3PDH) that were also measured in each sample with the following equation: relative target mRNA levels = [1/(1 + E_target_)^CT_target_]/[1/(1 + E_geomean_)^CT_geomean_] × 100, where E is the reaction efficiency and CT is the average cycle threshold of the duplicates [49,59,61]. Normalization to multiple reference genes, rather than a single gene, provides a more accurate quantification of mRNA levels [62,63]. Mean CT values for 18s and G3PDH did not differ between male phenotypes or among female groups (*p *> 0.05), demonstrating they are appropriate reference genes for the comparison of mRNA levels within a sex. However, when mean CT values were compared between sexes, those of 18s (Mann-Whitney test, *p *= 0.021), but not G3PDH (*p *= 0.092), differed between pooled males and females. For sex comparisons, we therefore normalized the target genes only to G3PDH.

### Steroid assays

Plasma testosterone (T), 11-ketotestosterone (11-KT), estradiol (E_2_) and cortisol (CS) were measured using commercially available Enzyme ImmunoAssay (EIA) kits (Cayman Chemical, Inc.). For CS, serum was directly diluted 1:40 in assay buffer prior to plating. For T, 11-KT, and E_2_, a 5 μl sample of plasma from each subject was extracted three times using 200 μl of ethyl ether and evaporated under a fume hood prior to re-constitution in assay buffer (1:40 dilution; extraction efficiencies 87-89%). EIA kit protocols were then strictly followed, plates were read at 405 nm using a microplate reader (UV_max _Microplate Reader, Molecular Devices), and steroid concentrations determined based on standard curves. All samples were assayed in duplicate, intra-assay coefficients of variation (CV) were: CS (7.3%, 10.6%); T (6.2%, 10.3%); 11-KT (4.9%, 3.1%); E_2 _(6.0%, 9.1%), and inter-assay CVs were: CS (5.7%); T (8.4%); 11-KT (4.2%); E_2 _(6.2%). Each EIA kit was validated by extracting steroids from a pooled *A. burtoni *serum sample and comparing that serially diluted sample curve to the standard curve for that particular hormone according to the methods described by Plikaytis et al. [64]. Parallelism was confirmed according to these guidelines for all four steroid assays: within-dilution CVs were all ≤ 15%, (CS, 1.2-8.2%;T, 6.4-9.4%;11-KT, 0.13-7.6%; E_2_, 0.0-7.9%), and within-assay CVs were all ≤ 20% (CS, 0.51%; T, 7.2%; 11-KT, 0.48%; E_2_, 6.5%). Absolute hormone values measured here were similar to those reported in previous *A. burtoni *studies that also used EIA kits [51,55,65].

### Statistics

Data sets that were normally distributed with equal variances were analyzed with Student's t-tests or one-way analysis of variance (ANOVA) with post-hoc Tukey's tests for multiple comparisons. Data that did not meet the assumptions of parametric statistics were compared with Mann-Whitney rank sum tests or Kruskal-Wallis tests (KW) with post-hoc Dunn's tests. For consistency however, all data are plotted as mean ± standard errors (SE) with appropriate statistical test values reported in the text. To test for sex differences in mRNA levels for each receptor subtype, data were also pooled within each sex and then compared with Mann-Whitney tests. Correlations were assessed with either Pearson product moment tests (parametric) or Spearman rank tests (non-parametric). Our results are presented and interpreted without the use of conservative corrections for multiple comparisons such as the Bonferroni correction. However, Bonferroni corrected *p*-values are also indicated in the correlation tables for reference (Bonferroni corrected *p *= 0.05/n, where n is the number of hypotheses tested on a set of data). While the use of Bonferroni and related procedures may reduce Type I errors, they also reduce statistical power and increase the chance of Type II error to an unacceptable level, especially in cases of smaller sample sizes (i.e., n < 30) [66]. We therefore chose to report observed effect size (e.g., *r *values) along with exact *p*-values to allow reader evaluation of biological importance, rather than utilize the overly conservative Bonferroni correction. Statistical comparisons were performed with SigmaPlot 11.0 (Systat Software, Inc., San Jose, CA.).

## Authors' contributions

KPM devised the study, collected and analyzed the data, and wrote the initial draft of the manuscript. RDF provided input on experimental design and data interpretation, and contributed to writing of the manuscript. Resources and laboratory space to conduct the experiments were also provided by RDF. Both authors read and approved the final manuscript.
